# Recent progress and new challenges in modeling of human pluripotent stem cell-derived blood-brain barrier

**DOI:** 10.7150/thno.63195

**Published:** 2021-11-02

**Authors:** Li Yan, Rebecca A. Moriarty, Kimberly M. Stroka

**Affiliations:** 1 Fischell Department of Bioengineering, University of Maryland, College Park, MD 20742, USA; 2 Biophysics Program, University of Maryland, College Park, MD 20742, USA; 3 Center for Stem Cell Biology and Regenerative Medicine, University of Maryland, Baltimore, MD 21201, USA; 4 Marlene and Stewart Greenebaum Comprehensive Cancer Center, University of Maryland, Baltimore, MD 21201, USA

**Keywords:** blood-brain barrier, pluripotent stem cells, brain microvascular endothelial cells, neurovascular unit, disease modeling.

## Abstract

The blood-brain barrier (BBB) is a semipermeable unit that serves to vascularize the central nervous system (CNS) while tightly regulating the movement of molecules, ions, and cells between the blood and the brain. The BBB precisely controls brain homeostasis and protects the neural tissue from toxins and pathogens. The BBB is coordinated by a tight monolayer of brain microvascular endothelial cells, which is subsequently supported by mural cells, astrocytes, and surrounding neuronal cells that regulate the barrier function with a series of specialized properties. Dysfunction of barrier properties is an important pathological feature in the progression of various neurological diseases. *In vitro* BBB models recapitulating the physiological and diseased states are important tools to understand the pathological mechanism and to serve as a platform to screen potential drugs. Recent advances in this field have stemmed from the use of pluripotent stem cells (PSCs). Various cell types of the BBB such as brain microvascular endothelial cells (BMECs), pericytes, and astrocytes have been derived from PSCs and synergistically incorporated to model the complex BBB structure *in vitro*. In this review, we summarize the most recent protocols and techniques for the differentiation of major cell types of the BBB. We also discuss the progress of BBB modeling by using PSC-derived cells and perspectives on how to reproduce more natural BBBs *in vitro*.

## Introduction

The blood-brain barrier (BBB) is a highly selective barrier that is comprised of endothelial cells and that separates the brain parenchyma from peripheral blood. This barrier restricts the access of hydrophilic molecules and leukocytes from the blood into the central nervous system (CNS), thereby playing a critical role in protecting neuronal tissue and maintaining brain homeostasis 1. The breakdown of the BBB leads to the entry of toxins and pathogens, as well as infiltration of immune cells into the CNS, subsequently contributing to neurological imbalance. BBB dysfunction is involved in the pathogenesis of many neurological diseases, including stroke, trauma, multiple sclerosis, Parkinson's disease (PD), and Alzheimer's disease (AD) 2. This is further compounded by the fact that the BBB is a major obstacle for the CNS drug delivery. Certain small lipid-soluble drugs with molecular weight under 400 Da may cross the BBB via transmembrane diffusion 3, but over 90% of CNS drugs are not transported across the BBB to enter the CNS, thus impeding the treatment of neurological diseases 4, 5.

The neurovascular unit (NVU) is considered to be a broader structural basis than the BBB and is used to describe the interplay between the blood and the brain. The NVU consists of brain microvascular endothelial cells (BMECs) connected by cell-cell junctions, which line the blood vessels and interact closely with an encapsulating layer of mural cells (pericytes and vascular smooth muscle cells (SMCs)), astrocytes, as well as other surrounding neural cells. Combined with the basement membrane, these cells form a dynamic system that maintains the cerebrovascular integrity and transduce biochemical and biomechanical signals to regulate the BBB's functions 6. The physical barrier system restricts the nonspecific transport of large and small molecules across the BBB. It is also the major limiting factor for the therapeutic drugs that aim to penetrate the brain. Despite these limitations, specialized endogenous transport mechanisms exist to enable the transcytosis of molecules entering the CNS to maintain the brain homeostasis. Vesicular-based transport systems such as receptor mediated transcytosis (RMT) have become long-standing strategies for the delivery of therapeutics and biologics to the CNS 3, 7.

Primary cell lines from brain tissues such as primary BMECs, pericytes, and astrocytes are widely used to construct the NVU and investigate the BBB function *in vitro*. However, these primary cells are usually isolated during a brain biopsy, leading to a high degree of batch-to-batch difference. Obtaining a large cell number and retaining BBB properties during proliferation *in vitro* are challenging. In culture, primary NVU cells lose some of their key phenotypes over several passages, and the expression of specific transporters, enzymes, and trans-endothelial electrical resistance (TEER) values are much lower than that of *in vivo* cells. As a result, the scalability of primary cells is limited. It is also not ethical or practical to isolate large amounts of primary cells from human patients, which limits the personalized or industrial BBB development *in vitro* 8.

Recent advances in the field of human pluripotent stem cells (hPSCs) make it possible to provide a robust and scalable cell source for BBB modeling 9-13. hPSCs derived from healthy and diseased patients can be an ideal alternative to primary cell or animal models for human diseases *in vitro* 14. hPSCs, including human embryonic stem cells (hESCs) and induced pluripotent stem cells (iPSCs), can differentiate into various NVU cell types such as BMECs, pericytes and astrocytes, which can be assembled into an *in vitro* BBB model. In addition to NVU cells, other cell types such as mesenchymal stem cells (MSCs) can also improve the stabilization of BBB 15, 16. Previous studies creating hPSC-BBB constructs proved to recapitulate the complex BBB function *in vitro,* such as well-formed tight junction protein expression which led to physiologically equivalent TEER values 17. Recent hPSC-BBB models also display various adhesion molecules that are suitable for the study of immune cell migration across the BBB 9. Due to the expression of proper transporter proteins, BBB models have been used for predicting permeability of CNS drugs 18. Thus, BBB models derived from hPSCs provide an important platform for high-throughput screening of brain-penetrating molecules 18. Furthermore, the isogenic personalized BBB models derived from patient-specific iPSCs can improve our understanding of the BBB in both physiological and pathological conditions 19.

In this review, we provide an overview of PSC-derived BBB models. We first detail the specialization of the BBB and discuss its unique characteristics. Then, we summarize the recent differentiation protocols for major cell types that contribute to barrier function. Next, we illustrate how these cell types are harnessed to engineer PSC-derived BBB models, including transwell, organoids and microfluidic BBB-on-chip systems. Finally, we discuss the personalized BBB modeling of CNS diseases and provide perspectives on how to improve the hPSC-derived BBB modeling *in vitro* and discuss critical questions in the field that require further investigation.

## 1. Structure and physiology of BBB

**Figure 1:**
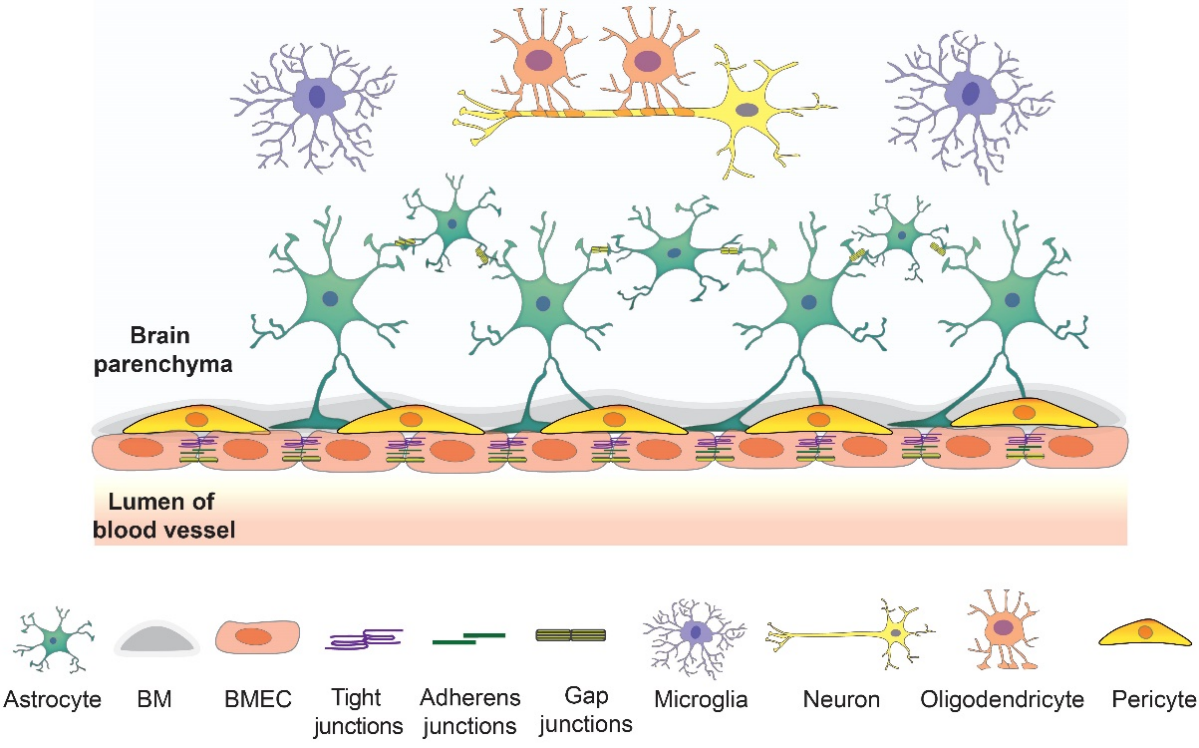
The overview of the components of NVU. BMECs connected by junction proteins intimate contact astrocytes and astrocytes in basement membrane creating a strong barrier. This barrier interacts with other brain cell types such as neurons, microglia, and oligodendrocytes to maintain the brain homeostasis.

The barrier layers of the CNS separate the blood and neural tissues, which synergistically contribute to maintaining brain homeostasis. There are three barriers in the brain, including the BBB, the blood-cerebrospinal fluid barrier, and the epithelial cells of the arachnoid membrane at the brain surface. The BBB is a vast network of over 650 kilometers of small capillaries, of which the average diameter is about 5-8 μm 20, 21. The NVU at the capillary level is composed of BMECs, astrocyte end-feet, and pericytes embedded in the capillary basement membrane, which also interact with surrounding neuronal cells (Figure 1). This dynamic system allows the flux of various molecules which is crucial to maintain normal brain function 6.

### 1.1 BMECs

BMECs are specialized endothelial cells lining the microvasculature of the CNS. BMECs are the main player in forming the physical, transport and metabolic barrier restricting the movement of molecules and immune cells between the blood and brain. A continuous sealed BMEC capillary gives rise to a complex and dynamic barrier which limits paracellular transport and diffusion of molecules, ions, and proteins and maintains apical-basal polarity 22. The properties of this barrier are determined by junctional complexes between adjacent BMECs comprised of tight junctions, adherens junctions, and gap junctions. Tight junctions consist of integral membrane proteins and several accessory proteins, such as Occludin, Claudins, junction adhesion molecules, and zonula occulden (ZO) proteins. These transmembrane adhesion proteins are arranged parallel to the direction of blood flow and link to the actin cytoskeleton to provide structural support that maintains the BBB integrity 23. Adherens junctions are composed of two families of transmembrane proteins: cadherins and nectins 24, 25. By cooperating with afadin and catenin, they form two structurally adhesive units: the cadherin-catenin complex and the nectin-afadin complex. Both mediate cell-cell adhesions and provide scaffolding for tight junction formation. The interactions between ZO-1 and catenin or afadin influence tight junction assembly and reinforce the barrier function 26. Gap junction intercellular communication is formed by two hemichannels which are composed of six transmembrane connexins proteins. Unlike tight and adherens junction proteins, connexins do not form a tight seal between the adjacent cells. Connexins mediate intercellular communication, which is regulated by pH, voltage, calmodulin and phosphorylation 27, and the opening of connexin hemichannels is controlled in a Ca^2+^-dependent manner 28. Due to these well-formed junctional complexes, BMEC monolayers have high TEER values; this differs from other peripheral endothelial cells. The physiological TEER value of brain capillary is >1000 Ω·cm^2^ which is much higher than 3-33 Ω·cm^2^ in other tissues 29.

### 1.2 Pericytes

Pericytes are branched cells present along the basal wall of capillary blood vessels throughout the body. Although pericytes are widely distributed throughout all organs of the entire body, the density of pericytes varies in different organs and vascular beds, and CNS vasculature is generally regarded as the most pericyte-covered district with an approximately 1:1 to 1:3 pericytes-to-endothelial cells ratio 30. In the CNS, pericytes play important roles in angiogenesis, neovascularization, maintenance of the BBB integrity and homeostasis, regulation of immune cell infiltration, and control of cerebral blood flow. Pericyte deficiency by mutants of platelet-derived growth factor-β (PDGFβ) impairs BBB function by increasing brain vessel permeability, causing abnormal polarization of astrocyte end-feet, and reducing astrocyte-derived basement membrane component Lama2 31. The communication between brain endothelial cells and pericytes plays an important role in maintaining the integrity of BBB. This communication is mediated by paracrine signals such as transforming growth factor beta, PDGF-BB, angiopoietin 2, and vascular endothelial growth factor. These factors also affect the survival and contractility of pericytes 32. In addition, the interaction of pericytes and astrocytes stabilize BBB function. Astrocytic laminin regulates pericyte differentiation from a contractile stage to a resting stage, and thus contributes to BBB integrity 33. Furthermore, apolipoprotein secreted by astrocytes binds to the receptor on pericytes altering BBB permeability 34.

### 1.3 Astrocytes

Astrocyte cells are a heterogeneous population with distinct morphology and function, depending on their specific brain regions in the CNS. They are the most abundant glial cell type in the CNS and play critical roles in the formation and modulation of functional synapses, elimination of toxins and debris, and maintenance of BBB coupling with endothelial cells and pericytes 35. Astrocytes encircle the vasculature of the CNS with their end feet, which anchor the subendothelial basal lamina and secrete a complex array of biomolecules in the basal lamina influencing endothelial cells and other surrounding cells. A highly specialized astrocyte-endothelial interface is established through inductive processes and direct interaction. Endothelial cells promote the water channel, aquaporin-4 (AQP-4), accumulation in astrocytes' endfeet by compounds they secrete in the extracellular matrix (ECM), such as agrin, as well as direct mechanical interaction with the end feet 36. Gap junction-mediated intercellular communication is also a typical feature of astrocytes. The connexin gap junction channels on astrocyte end feet mediate the cross-talk between different counterparts of the BBB via Ca^2+^ signaling 27. Astrocytes also have the ability to respond to and regulate brain inflammation. Astrocytes acquire different phenotypes when they react to different pathological stimuli. The proinflammatory astrocytes secrete pro-inflammatory factors that increase BBB permeability and leukocyte infiltration, which are particularly related to the brain pathologies of diverse neurodegenerative diseases 37, 38. In contrast, the newly formed reactive astrocytes in glial scars repair the BBB and restrict the inflammatory cells entering the injury or stroke site within the CNS 39. In addition, region-specific astrocytes, especially in white matter and grey matter, show extensive functional differences, such as different expression of transporters and responses to stimuli, which affect the BBB phenotypes and contribute to BBB heterogeneity 40.

### 1.4 Basement membrane

The basement membrane surrounding the vascular tube is a unique ECM supporting the BMECs, pericytes, and astrocytes. The basement membrane exerts important functions by maintaining structural specificity and membrane stability. It provides an anchor for many signaling processes at the vasculature interface, but also provides an additional diffusion barrier for molecules and cells to cross before accessing the neural tissue. The basement membrane is a 30 to 40 nm lamina comprised of secreted molecules including fibronectin, nidogen, type IV collagens, laminin, heparin sulfate proteoglycans, and other glycoproteins. The composition of these ECM proteins changes in the microenvironment of the BBB at different development stages or disease 41. Meanwhile, disruption of the basement membrane increases BBB permeability and leads to leukocyte infiltration and BBB dysfunction 42.

### 1.5 Transport system of BBB

Due to the presence of a specialized junction network, BMECs effectively restrict free paracellular transport. They express designated transporters and allow extremely low rates of transcellular transport, exerting a higher degree of control to molecular, metabolite, and nutrient exchange across the BBB 43. The BBB barrier is highly permeable to gaseous molecules such as O_2_, CO_2_, N_2_, H_2_O, as well as volatile anesthetics. Small lipophilic molecules can pass the barrier by diffusing across the lipid bilayer membranes along their concentration gradient 44. Movement of all other molecules across the BBB is dependent on the presence of transporters. Glucose, as the primary energy substrate, enters the brain by carrier-mediated transport, glucose transport protein type 1 (GLUT-1) 45. Large molecules, such as peptides and proteins, enter the brain by adsorptive mediated transcytosis (AMT) or RMT 46. Smaller peptides cross the BBB by either nonspecific endocytosis or RMT. BBB receptors such as transferrin receptor (TfR), insulin receptor, lipid transporters (e.g. low-density lipoprotein receptor), solute carrier family transporter, and leptin receptor recognize the circulating protein ligands and transport the bounding ligands into brain parenchyma via RMT 46, 47. Drug efflux transporters such as P-glycoproteins (Pgp),multidrug-resistance related protein (MRP), and breast cancer resistance protein (BCRP) can actively transport a huge variety of lipophilic drugs out of the cells forming the BBB which limits the access of drug to CNS tissues 48.

## 2. Development of the BBB

The barrier properties of brain tissue were first demonstrated by the studies of Ehrlich, Franke, Bouffard, and Goldman around the early 1900's. They showed trypan red, methylene blue, and trypan blue can stain all tissues except the nervous system, which indicates that the barrier system existing in the brain and the CNS is unique from the rest of the body 49, 50. Stern and Gautier reported detailed studies of penetration of a wide range of molecules from blood into brain and proposed the concept of a BBB in 1921 49. Thereafter, many dyes and low molecular weight compounds were used to study BBB permeability by various routes in different species from embryonic stages through adulthood 50. Grazer and Clemente injected trypan blue into rat embryos from embryonic day 10.5 (E10.5) to birth and found no staining of brain tissue 51. Similar results were reported by intravascular injection of fluorescently labeled bovine albumin in rat embryos at E15 52. This collective evidence demonstrates that BBB formation begins during early embryonic stage. The permeability experiments using small molecules (e.g., sodium ferrocyanide) suggested that the BBB in young animals is leakier and more permeable than in adult animals. Some interpretations of previous data argue that the BBB in embryos, fetus, and neonate is less mature compared with adult 50. However, interestingly, there has been recent controversy over the degree of maturity and permeability of the developing BBB 49. The BBB during development displays stage-specific properties 53, and different transporters of the BBB are developmentally regulated 54. For example, protein levels of ABCB1 were higher at E13 than in the adults 53. Solute-linked carrier transporters were expressed at higher levels in the fetal choroid plexus compared to that of adults 50. Mannose 6-phosphate/insulin-like growth factor 2 receptor had high fetal and prenatal levels, followed by decreased postnatal levels 55. These reports show that higher expression of some transporters is necessary to facilitate rapid nutrient diffusion or prevent the toxin from brain, which may indicate leaky properties of the BBB during early stages is actually more indicative of the functional specialization 49.

In mammals, the specific properties of the BBB are induced during CNS angiogenesis. The development of the brain vascularization begins with angiogenic sprouting from a perineural vascular plexus. The capillaries sprout and invade the neuroepithelium and form a functional vascular network 56. Meanwhile, the vascular progenitor cells grow into the embryonic neuroectoderm 26. In the rat cerebral cortex, neuronal angiogenesis begins at E12, approximately halfway through the normal 24-day gestation time. Neural progenitor cells secrete molecular signals such as vascular endothelial growth factor (VEGF) and Wnt that guide the migration of endothelial progenitor cells into the neural tissue from the surrounding vascular plexus towards the ventricles 57, 58. In mice, the early BBB properties begin at stage E11, including elevated expression of tight junction proteins and efflux transporters, and downregulation of transcytosis (plasmalemma vesicle-associated protein) and leukocyte adhesion molecules (ICAMs) 54. The expression of GLUT-1 gradually decreases in neuroepithelial cells and increases in the brain endothelial cells from E12 to E16 in rat brains to meet the increasing demands for glucose 59. ATP-binding cassette (ABC) efflux pumps such as ABCC and BCRP are expressed at E12.5 in mouse and show apparent change in expression throughout development 60, while Pgp is expressed at low levels during embryogenesis and increases during postnatal development 54. This indicates that different transporters have different regulation mechanisms during development 60. The fenestrated parenchymal vessels at E13 and low TEER indicate an incomplete barrier function with high permeability 61. The maturation of the BBB starts from E13 in intraparenchymal vessels and E17 in pial vessels. At these time points, the fenestration declines rapidly. The TEER value was found to be increased significantly in pial vessels of the rat at E21 62. Further analysis in mice indicates tight junction molecules occludin, claudin-5 and ZO-1 are expressed at E12 54. The junctional strands are visible in brain capillaries beginning at E13.5 and the BBB becomes tightly restricted between E14.5 and E15.5 63, 64. In human brain, tight junction proteins occludin and claudin-5 were first detected at the interface of adjacent endothelium at 14 weeks gestation 65.

During the invasion process, endothelial cells secrete factors such as PDGFβ to recruit pericytes, which are critical for tight junction formation. The BBB forms during endothelial cell invasion and pericyte recruitment to the nascent vessels. The evidence has demonstrated that pericytes are not required for induction of BBB-specific genes but are vital to control the relative permeability of CNS during embryogenesis 54, 64. In early embryogenesis, neural progenitor cells connect with endothelial cells and pericytes to promote BBB maturation. Neural progenitor cells induce angiogenesis and BBB gene expression via the Wnt signaling pathway 54, 57. Therefore, it is clear that many BBB properties are induced and well formed during early embryogenesis. Another cell type essential for development and maintenance of the BBB is astrocytes. Although astrocytes were first detected in the cerebral cortex at the late embryonic stages around birth, the major differentiation and production of astrocytes occurs during the first month of the postnatal period, suggesting that astrocytes contribute to BBB maturation and maintenance instead of induction of BBB formation 54, 57.

## 3. hPSC-derived BBB cells

BMECs, pericytes, and astrocytes are the three major cell types forming the BBB and function to tightly regulate the exchange of substances between the blood and the brain tissue. hPSCs could provide renewable and reproducible sources of these cell types at relatively low costs compared to primary cells. To mimic the *in vivo*-like BBB function, development of reliable and cost-effective differentiation protocols of these cell types is very crucial. Herein, we discuss the representative protocols for BMECs, pericytes, and astrocyte differentiation from PSCs.

### 3.1 hPSC-derived BMECs

hPSCs, including both hESCs and iPSCs, have previously been differentiated to endothelial cells. PSC-derived BMEC-like cells are identified by expressing tight junction proteins and possessing BBB-like properties, such as low passive permeability, high TEER, and active efflux transporter and RMT functions^66^. The first protocol for differentiation of BMEC-like cells was reported in 2012 67. An unconditioned medium (UM) containing Medium/Nutrient Mixture F12 (DMEM/F12), knockout serum replacement, nonessential amino acids, Glutamax, and β-mercaptoethanol was used to initiate co-differentiation to neural and endothelial progenitors within 7 days. The GLUT-1^+^PECAM1^+^ endothelial population is elevated to the predominant cell type by expanding the cells in endothelial cell (EC) medium. Subculture of ECs on fibronectin-collagen type IV matrix is critical for purification and maturation of BMEC-like cells (UM BMEC-like cells). The barrier properties were determined by TEER measurements and active transporter function (Pgp, BCRP, and MRP). Cocultures of UM BMEC-like cells with rodent astrocytes elevated the TEER value from 150-170 Ω·cm^2^ to a maximum of 1,450 ± 140 Ω·cm^2^ and showed expression of a variety of receptors and transporters 67. During BMEC differentiation, retinoic acid (RA) was shown to trigger several modes of action and boost the passive barrier properties of hPSC-derived BMEC-like cells 68. RA-treated UM BMEC-like cells (UM-RA BMEC-like cells) exhibited a maximum TEER value of 5350 ± 250 Ω·cm^2^ when cocultured with human primary pericytes and neural progenitor cells in a modified EC medium. However, the TEER value dropped dramatically after 3 days and a large variation was found between UM-RA BMEC-like cells differentiated from different cell lines. The tested function of three efflux transporter families (p-glycoprotein, BCRP, and MRP) increased only MRP expression and activity after RA treatment 68.

E6 medium is a fully-defined and xeno-free medium that was used to replace unconditioned medium to regulate iPSC-derived BMEC-like cell specification by inducing iPSCs to neuroectoderm 69. After 4 days treatment with E6 medium, immature BMEC-like cells were switched to human endothelial serum-free medium (hESFM) supplemented with basic fibroblast growth factor (bFGF), RA, and platelet-poor plasma-derived bovine serum (PDS). BMEC-like cells are purified by subculturing cells on fibronectin-collagen type IV matrix with EC medium without bFGF and RA. BMEC-like cells generated with this protocol (E6 BMEC-like cells) have been shown to have equivalent paracellular permeability and efflux transporter activity compared to the UM-based method and maintain TEER values above 1000 Ω·cm^2^ for at least 8 days in monoculture 70. Later, a modified defined protocol arose based on the E6 BMEC-like cells, but replaced PDS with fully defined factors (N2, B27, or ITS) to provide a cost-effective approach to generate BMEC-like cells with more stable TEER values 71. Neurobasal medium and DMEM/F12 were used to replace the hESFM to study the BBB properties under completely defined culture conditions. The basal media change influenced the gene expression of various transporters and the activity efflux transporter in hPSC-derived BMEC-like cells 72. In addition to an optimal media composition, the initial cell density of differentiation is crucial for the PSC-derived BMEC-like cells to achieve marked barrier function. For example, in one study, 3.5×10^4^ cells/cm^2^ was the optimal seeding density to obtain uniform junction protein expression and high TEER value 73.

Prior to BBB establishment, human brain development in early embryos was purported to occur in a hypoxic environment. Hypoxic conditions have been found to enhance the BBB properties of UM BMEC-like cells, such as ECM deposition, TEER value, and the activity of ABC transporters Pgp, MRP1&4, and BCRP. Hypoxia induced BMEC-like cells also exhibit proper transcellular transport of drugs, peptides, nanoparticle, and antibodies that are dependent on TfR and lipoprotein receptor-related protein. The expression level of Wnt7a also increased 25-fold compared with normoxic conditions 74. It is important to note that canonical Wnt-β-catenin signaling is necessary for induction of brain angiogenesis. Wnt-β-catenin signaling induces mesodermal and endothelial commitment. Wnt7a and Wnt7b promote BBB specification of UM BMEC-like cells 67. The canonical Wnt pathway agonist, CHIR99021, directs differentiation of hPSCs to BMEC-like cells through an intermediate primitive streak stage. The subsequent RA treatment leads to BMEC-like cells (CHIR-RA BMEC-like cells) with BBB properties 73. Notably, RA has been reported to induce endothelial immune quiescence by preventing the brain endothelium from expressing IL-6, CCL2, and vascular cell adhesion molecules (VCAM) 75. Similar results showed that UM BMEC-like cells and CHIR-RA BMEC-like cells lack vascular cell adhesion molecules (ICAM-2, VCAM-1, E-selectin, or P-selectin) necessary for immune cell adhesion and trafficking 9. An extended EC culture method without RA (EECM) was developed to generate BMEC-like cells (EECM BMEC-like cells). Despite low TEER value and permeability to sodium fluorescein, EECM BMEC-like cells express ICAM-1 and VCAM-1 which increase the Th1 cell adhesion under cytokine stimulated conditions 9.

Notably, hPSC-derived BMEC-like cells differentiated by most current methods lack some phenotypic and functional features of bona fide ECs 76-78. Transcriptomic analysis has shown underlying epithelial-like gene expression in hPSC-derived BMECs 76, 79. Recently, there has been a controversy on the identity of PSC-derived BMEC-like cells. Lu, *et al*. employed a comprehensive transcriptomic metanalysis of the hPSC-derived BMEC-like cells generated by current protocols and found that many current protocols produced a homogenous epithelial cell population (including UM 67, UM-RA 68, E6 70, and defined medium induced BMEC-like cells 71, 73) lacking vascular endothelial identity 76. They termed these cells as epi-BMEC-like cells. From their work, overexpression of endothelial ETS factors (ETV2, FLI1, and ERG) in UM induced BMEC-like cells at D6 directs more EC phenotypes. BMECs derived by this method harbor EC transcriptomic profiles, express EC markers (PECAM1, CDH5, KDR), respond to inflammatory stimuli, and display angiogenic properties via tube formation assays in mouse 76.

### 3.2 hPSC-derived pericytes

The heterogeneous distribution and function of pericytes makes it difficult to accurately distinguish them from other related cell types, such as SMCs or MSCs 83. No specific markers are known to be unique for their identification. Generally, criteria of multiple markers are applied to isolate and define pericytes; these markers include contractile and cytoskeletal proteins (e.g., desmin, α-smooth muscle actin (α-SMA)) and cell surface antigens (e.g., platelet-derived growth factor receptor β (PDGFRβ), transmembrane chondroitin sulfate proteoglycan (NG2), regulator of G-protein signaling-5) 34, 84. Additionally, these markers also vary within different microvascular zones and developmental stages. Pericytes along the arteriole end of the capillary bed express more α-SMA. In the middle of the capillary bed, pericytes express less α-SMA, and capillary pericytes are α-SMA negative 85, 86. *In vivo* lineage tracing has revealed that CNS pericytes originate from both mesoderm and ectoderm depending on their exact location. Quail-chick chimera studies have shown that neural crest cells form pericytes in forebrain, while cells of the mesoderm form pericytes in the brainstem, mid-brain, and spinal cord 87, 88. Lineage tracing in mouse models has shown that capillary pericytes and vascular SMCs in retina, optical nerves and CNS are derived from neural crest 89.

As mesoderm and neural crest are two major origins of pericytes, pericytes have been derived from hPSCs from both starting points. One approach used embryonic bodies (EBs), cultured in serum through an intermediate multilineage stage containing mesoderm and neuroectoderm. From the EBs, the isolated subset of mesodermal precursors (CD105^+^ CD31^-^ cells) gave rise to CD146^+^NG2^+^PDGFβ^+^SMA^-^ pericytes 90. Upon mesoderm to endothelial cell induction, pericytes can be derived from the CD31^-^ fraction by a fully defined protocol 91, 92. VEGF and SB431542 induce hPSC differentiation toward early vascular cells (EVCs), which can give rise to both endothelial cells and pericytes. Pericytes can be generated from a CD34^-^ population while simultaneously generating endothelial progenitor cells 93. VEcad^-^ cells sorted from EVCs have the capacity to differentiate to NG2^+^PDGFRβ^+^CD44^+^ pericytes 94, 95.

Meanwhile, coculture of hPSC and OP9 stromal cells induce APLNR^+^PDGFRα^+^ primitive posterior mesoderm. FGF2 directs APLNR^+^PDGFRα^+^ primitive posterior mesoderm to mesenchymoanioblast precursors, which have the potential to generate SMCs, pericytes, and MSCs. Pericytes can be further specified to CD274^+^ capillary and delta like homolog 1 positive arteriolar pericytes, which exhibit a proinflammatory or a contractile phenotype, respectively 96. Pericytes differentiated from mesoderm are usually found with simultaneous endothelial differentiation. However, neural crest cells were reported to contribute to pericytes and SMCs rather than CNS endothelial cells. Activating Wnt signaling and inhibiting bone morphogenetic protein 4 (BMP4) and activin/nodal signaling induce neural crest stem cell specification. By following culture in a serum contained medium, NSCs can be differentiated to pericytes 97, 98. Based on these methods, *in vivo* integration, vasculature formation, and co-culture with endothelial cells have been used to confirm pericyte functions (Table 2).

### 3.3 hPSC-derived astrocytes

Given the significance of astrocytes in CNS function, various protocols have been developed to direct differentiation of hPSCs toward different subtypes of astrocytes (Table 3). Generally, astrocyte differentiation is initiated from an induction of neural progenitor cells, and then followed by astrocyte specification and maturation. Neural progenitor cells (NPCs) are commonly induced in EBs by modulating SMAD signaling pathway 101. The NPCs in both EB culture or monolayer culture can be initiated and expanded by addition of a cocktail of small molecules (e.g., SB431542, dorsomorphin, noggin) and growth factors (e.g., bFGF, EGF). NPC induction is confirmed by the appearance of neural rosettes and the presence of NPC markers such as Pax6 and Nestin. After neural induction, astroglial specification is regulated by a combination of various developmental morphogens such as LIF, CNTF, SHH, BMP, and RA. To characterize the hPSC-derived astrocytes, the presence of glial fibrillar acidic protein (GFAP) has been considered as the gold standard for identifying astrocytes. Meanwhile, S100β is a widely used marker that is expressed in astrocyte progenitors. AQP-4, glutamine synthetase, glutamate transporter-1, and glutamate aspartate transporter (GLAST-1) are also used to identify astrocytes at different stages of differentiation 13. In addition to specific markers, several assays are available to characterize the functions of hPSC-derived astrocytes. These include analysis of glutamate uptake, calcium signaling, electrophysiological properties, and synapse formation. Promoting the maturation of hPSC-derived astrocytes is challenging due to the heterogenous morphology and function of astrocyte subtypes in different brain regions. Usually, the protocols for astrocyte differentiation are technically complicated and require long-term culture. The astrocyte yield from these protocols is a mixture of cells at different differentiation stages combined with a lack of regional specification. Recently, hPSC-derived astrocytes show region-specific phenotypes associated to dorsal and ventral forebrain or dorsal and ventral spinal cord 102.

## 4. Advances in hPSC-derived BBB modeling

**Figure 2:**
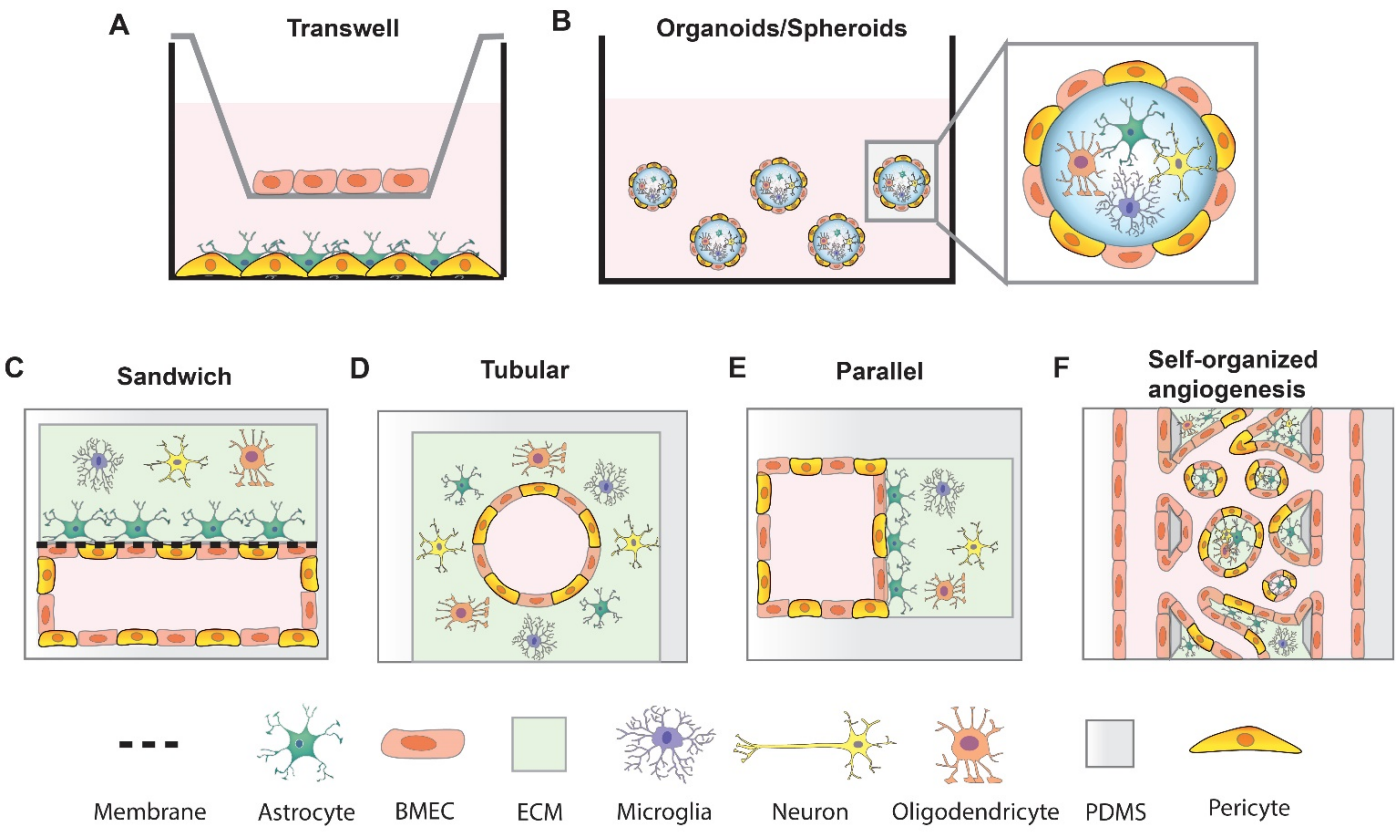
Strategies of *in vitro* BBB models. (A) transwell BBB model; (B) BBB spheroid and organoid model; (C) Sandwich BBB model; (D) Cylindrical tubular BBB model; (E) Parallel BBB model; (F) Self-organized BBB model.

*In vitro* models that mimic BBB function are crucial tools for studying neurological diseases and developing and testing brain-permeable drugs for clinical use. An ideal BBB model would be fully isogenic from a single source and exhibit robust BBB function with long-term stability. Such a high-fidelity BBB model would increase the efficiency of brain drug screening and boost the development of neurotherapeutics. To date, although reproducing key BBB features *in vitro* remains challenging, many researchers have developed different systems to model the BBB by incorporating hPSC-derived BMEC-like cells, pericytes, astrocytes and neuronal cells (Figure 2). These BBB models have proven useful for studying pathological dysfunction and predicting drug permeability. Thanks to the recent advanced techniques, sensitive and quantitative methods have been established to assess the functions of BBB models.

### 4.1. Functional assessment of BBB models

#### 4.1.1 Junction integrity and coverage

The spatial network of junction proteins in BMECs plays a vital role in stabilizing the BBB function. Immunostaining of both tight junction proteins, such as ZO-1, claudin-5, occludin, and adherins junction proteins, such as VE-cadherin, is commonly used to characterize the barrier integrity of BBB. Disruption of junction proteins revealed by the discontinuous or vanished junction patterns is a characteristic of BBB dysregulation and a hallmark of a number of CNS diseases. Coverage of continuous junction proteins can be quantified to indicate the changes in barrier properties in different conditions. Several methods have been reported to quantify junction integrity. Automated junction analysis by macros or plugins developed with ImageJ have been used to automate and streamline the evaluation of junction organization in cultured cells and the tissue sections 82, 111. A custom Matlab script was also reported to analyze the junction coverage in iPSC-derived BMEC-like cells 71. Recently, a Python-based Junction Analyzer Program (JAnaP) was developed by our lab to quantify perimeter junction protein phenotypes along with cell morphological parameters and has been published on GitHub for use by the wider scientific community 112. Using this program, we have evaluated BMEC junction architecture in response to different matrix stiffnesses, substrate composition, tumor cell-secreted factors, and photodynamic priming for drug delivery applications 112-115. For 3D vascular structures, a customized UNWRAP application has been developed to convert the cylindrical images to 2D planes 111, and we anticipate that this software can be integrated with the JAnaP to facilitate the quantitative analysis of 3D vessels.

#### 4.1.2 TEER

TEER is a widely accepted quantitative technique for measuring the integrity of tight junctions within a cell monolayer system. In this section, we first introduce the merits of TEER measurements for BBB models, but below we assert concern in utilizing TEER as a gold standard for comparing and assessing these models. The measurement of electrical resistance across a BMEC monolayer can be very sensitive and in general reliably indicates the integrity and permeability of cell monolayers 116. Conventionally, TEER measurements are performed in a transwell plate in which the BMEC monolayer is cultured on a semipermeable filter insert. The most widely used commercial TEER measurement system is known as an Epithelial Voltohmmeter that comes equipped with a pair of chopstick electrodes which have been applied in many studies. For electrical measurements, the shorter electrode is placed in the upper insert medium and the longer in the bottom well medium, and so they are separated by the BMEC layer and the transwell mesh support 67, 70. Compared to the physiological TEER (1500-8000 Ω·cm^2^), human primary BMECs have shown limited success in recapitulating the physiological TEER *in vitro,* consistently reporting values below 500 Ω·cm^2^ 1. Recently, human iPSC-derived BMEC-like cells have been reported to produce TEER values up to 5000 Ω·cm^2^ and show superior junction properties compared with primary BMECs. Coculture of hPSC-derived BMEC-like cells with pericytes, astrocytes or other neural cells can also boost the TEER value and enhance the barrier function. Despite the accepted validity of the TEER readings, in the transwell system, TEER values are not entirely stable, and they are highly dependent on the temperature, medium and electrode position within the wells. To avoid the variation of measurements with the chopstick method, a chamber system with fixed electrode geometry has been developed to generate more uniform results. The above-described systems and values for TEER measurements are mostly confined to static models 89. However, the physiological shear stress directly influences the barrier function and TEER value. For microfluidic BBB-on-chip systems, the TEER microelectrodes can be directly integrated into the chip system so that the electrodes are inserted to the pre-molded location on each side of the membrane-supported BMEC layer. As a result, TEER measurements can be continuously monitored for long-term study compared to conventional culture systems 117. Indeed, TEER continues to be a useful tool for measuring cell barrier integrity; however, the BBB community should also carefully consider whether a particular BBB model is also displaying other important BBB phenotypes when evaluating the fidelity of the model.

#### 4.1.3 Permeability

Under physiological conditions, limited permeability of the BBB restricts substances from the blood to the brain, which protects the brain from exposure to molecules that are toxic to the CNS 49. BBB permeability measurements as a metric for assessing BBB functions are important to understand the disease progression as well as evaluating therapeutic outcomes 118. Various dyes (e.g. Evans blue, trypan blue), radiolabeled proteins (e.g. albumin), and other markers (e.g. horseradish peroxidase, sucrose, dextran, sodium fluorescein) have been used to study the permeability of the BBB *in vivo* and *in vitro* and can reflect the different paracellular and transcellular transport mechanisms through the BBB 119. Fluorescently tagged dextran of varying molecular weights (ranging from 3 to 2,000 kDa) is a commonly used imaging marker for BBB permeability. Because of the wide range of molecular sizes, dextran can be used to test solute, ion, and protein permeability. In short, the fluorescent dextran is applied onto a cell monolayer and allowed to permeate through the cells (via either the paracellular or transcellular pathway), and then flow-through is measured on the other side of the cells (e.g., in a transwell). To precisely capture the integrity of the cell monolayer, small molecules are also available for studies of barrier permeability 120. Small molecule dyes such as sodium fluorescein (376 Da) and radiolabeled sucrose (342 Da) have been reported to effectively indicate the BBB integrity. With molecular weights of < 500 Da, they may enable detection of more subtle variations in BBB permeability when compared to the use of dextran 119. Sodium fluorescein and radiolabeled sucrose have been routinely tested on hPSC-derived BMEC-like cells in transwell assays, but they have not been widely used in microfluidic BBB models. Instead, dextran seems to be preferred in microfluidic BBB models. The quantitative measurement of BBB permeability in rodent brain showed that the diffusion values of the small solutes (sodium fluorescein and dextran) were between 3.3×10^-7^ cm/s and 4×10^-7^ cm/s 121, 122. hPSC-derived BMEC-like cells exhibit high correlation to the *in vivo* brain permeable values 67, 68, 70, 71, 73, 123. More recently, our lab has used a local permeability assay that was first described by Dubrovskyi *et al* 124 but modified by our lab to incorporate a biotinylated-fibronectin substrate seeded with human BMECs 98, and with FITC-avidin added to the cell culture media. Using this assay, we demonstrated a quantitative correlation between junction phenotype and local permeability 114.

#### 4.1.4 Transporter activity

BBB cells express a broad range of transporters that regulate entry of circulating chemicals into the brain by passive transport, the most well-studied of which are the ABC efflux pumps (Pgp, BCRP, and MRPs) 125. The most common and effective method to confirm activity of efflux pumps is to perform permeability experiments with inhibitors blocking the function of target efflux pumps in the presence of a specific substrate. For example, Pgp inhibitor (cyclosporin A, tariquidar, reversine, or verapamil) is used to test the Pgp function in BMECs by measuring the permeability of Rhodamine 123 which is a Pgp substrate. Treatment of Ko143 (a BCRP inhibitor) or MK571 (a MRP family inhibitor) results in increased accumulation of doxorubicin (a BCRP substrate) or 2′,7′-dichlorofluorescein diacetate (an MRP family substrate) 67, 68. The low rate of transcytosis is another important property that maintains the restrictive quality of the BBB. *In vitro* assays of transcytosis have been developed to screen antibodies and ligands and evaluate their therapeutic affinity and capacity 46. RMT receptors such as low-density lipoprotein receptor-related protein (LRP1), TfR, and insulin receptors abundant in brain capillaries have been exploited to increase the delivery of biotherapeutics to brain 126. LPR1 has been shown to bind to a variety of ligands such as aprotinin, apolipoprotein E, and lipoprotein lipase etc 127. Agiopep-2 (ANG) containing 19 amino acids is the well-known ligand to LPR1. Fluorescently labeled ANG has been used to target LPR1 for evaluating the RMT of BBB 128, 129. Artificial LPR1-binding ligands such as L57 130 are being researched for LPR-based drug delivery. The iron binding protein, transferrin, is the natural ligand for TfR. Fluorescently labeled transferrin can be used to test the RMT of BBB. Transferrin ligands and antibodies recognizing the TfR have been used for brain targeting 126, 131. Like the TfR, antibodies capable of binding to insulin receptor have been developed for BBB permeability 126.

### 4.2 Transwell-based BBB models

Transwell systems are the most commonly used and convenient *in vitro* model for BBB studies (Figure 2A). The low cost, ease of use, wide commercial availability, and flexibility of manipulating experimental conditions makes this apparatus a very robust tool to evaluate BBB properties. In transwell models, endothelial cells are usually cultured on the microporous semi-permeable inserts to form a monolayer on the apical side, while other cell types such as pericytes, astrocytes, or neural progenitor cells are cultured on the lower compartment forming the basolateral side. A range of pore sizes and different membrane compositions are available to satisfy diverse experimental requirements. Transwell-based BBB models with hPSC-derived BMEC monolayers have shown key features of BBB activity such as expression of tight junction proteins, transporter activity, and high TEER values. Incorporating UM-RA BMEC-like cells with pericytes, astrocytes, and NPCs enhances the BBB barrier function 68. Whole genome expression profiling confirmed the improvement in coculture over a monoculture system 79. Lippman *et al*. reported that UM BMEC-like cells cocultured with rodent astrocytes in a transwell system elevated TEER levels for 8 days compared with the TEER from BMEC monolayer, which dramatically decreased within 48 hours 67. UM-RA BMEC-like cells cocultured with primary human pericytes and neural progenitor cells yielded a significant increase of BMEC monolayer tightness and achieved a TEER value over 5000 Ω·cm^2^ 68. To improve the cell-cell contact, diverse coculture sets with primary or PSC-derived cells have been established based on the transwell system. For example, pericytes and astrocytes have been placed at the bottom surface of the transwell inserts 132. A 3D printed electrospun poly(lactic-co-glycolic) acid nanofibrous mesh replaced the transwell membrane to remodel the iPSC-derived BMEC and astrocytes interaction 133. The transwell model is also an important tool to identify matrix compositions and mimic ECM mechanics for BBB formation and maintenance. In another study, biodegradable substrates of varying composition were fine-tuned in a transwell-based BBB model to affect barrier function^134^. Collagen I gel is widely used to prepare hydrogels with low stiffness in BBB models. Coating collagen I hydrogels with basement membrane proteins such as collagen IV, fibronectin, and laminin improves UM-RA BMEC-like cell adhesion and proliferation. In contrast, coating collagen I gels with perlecan leads to poor adhesion of BMECs. Interestingly, although agrin promoted the adhesion of UM-RA BMEC-like cells, the TEER values of iPSC-derived BMEC on agrin-coated membranes were extremely low 134.

Transwell systems can be easily used for permeability screening of molecules or drugs by adding test solute to one side of the porous membrane, and solute concentration is then measured in the opposing well over time. UM-RA BMEC-like cells cultured with hiPSC-derived neural stem cells, pericytes and astrocytes show robust comprehensive transcellular drug transport 132. Patient-specific isogenic BBB models comprised of iPSC-derived BMEC, pericytes, and astrocytes established using transwell systems provide a valuable platform for neurovascular pathological study and drug discovery 135. However, this system has several well-known limitations. First, they are 2D monolayer systems that are unable to recapitulate key characteristics of the BBB. BMECs, pericytes, astrocytes or other cell types can only be applied on a flat geometry which lack the complex 3D cell architecture and limit the functionally-relevant cellular contacts. Second, the brain is one of the softest organs in the body. The flat substrates of a transwell membrane are much stiffer than native basement membrane ECM, which influences the cell-cell and cell-matrix signaling 136. Although 3D gel transwell systems have been developed to extend the geometry, the limited interaction between cells results in poor BBB properties 134. In addition, physiological shear stress is missing in this system, which can compromise barrier functions and leads to a model that fails to recapitulate the true *in vivo* environment 137. Thus, pathophysiological vascular diseases such as cerebral hypoperfusion and ischemia cannot be reproduced in this system.

### 4.3 Organoid and spheroid BBB models

An organoid is a three-dimensional miniaturized *in vitro* organ that is constructed by pluripotent or adult stem cells from various tissues. BBB organoids consist of multiple cell types that self-assemble in low-adherence culture conditions into multicellular constructs 138. The cells in BBB organoids and spheroids (Figure 2B) reproduce many BBB features, including high levels of tight junction proteins, an active efflux system, and specific molecular transporters due in part to the wealth of cell-cell contacts in this system 139-142 . The spheroids generated by primary BMECs, pericytes, and astrocytes exhibit higher expression of tight junction proteins, lower paracellular permeability, and higher drug efflux activity compared to a transwell co-culture system 142. A recently developed cortex organoid model contains six cell types, with BMECs and pericytes encapsulating the organoid generated from iPSC-derived astrocytes, oligodendrocytes, microglia, and neurons. This model recapitulates the various BBB functions and can maintain high cell viability for 21 days 141. Therefore, an important advantage of iPSC-derived organoid and spheroids models is that multiple cell types can be introduced into this model to more closely recapitulate the intricate NVU. The improved BBB function allows for more understanding of mechanisms of disease modeling and can evaluate the drug action for personalized medicine. However, organoid systems are limited in their size and long-term culture as a result of oxygen diffusion issues into the center of the organoid. The necrotic center has been observed in turn with cerebral organoid maturation 143. Although a previous study showed a prolonged duration of BBB organoid culture 141, the maintenance of barrier properties is still unclear at these later stages. Moreover, the TEER measurement and incorporation of applied shear stress are challenging in organoid and spheroid models.

### 4.4 Microfluidic BBB models

The BBB-on-chip systems can mimic the cellular microenvironment by precisely controlling niche factors such as 3D vessel-like structure, cell-cell interactions, cell-ECM interactions, substrate stiffness, and mechanical shear stress. The microfluidic BBB models that have addressed these characteristics have overcome limitations of other conventional BBB models (transwell or organoids) in aspects of 3D vascular structure and perfusion, live-cell imaging of permeability, and real-time monitoring of TEER value. A number of strategies have been explored to construct microfluidic BBB devices (Table 4, Figure 2C-F).

#### 4.4.1 Microfluidic BBB-on-chip

The classic on-chip model of the capillary comprises two polydimethylsiloxane (PDMS) microchannels which are separated by a porous membrane, giving the overall device the resemblance of a sandwich (Figure 2C). This model has been developed to create a variety of organ models, including (but not limited to) lung 144, gut 145 and BBB. In the BBB models, the porous polycarbonate (PC) membrane is coated with ECM proteins (collagens and fibronectin). BMECs are seeded in the ECM coated channel and brain cells are seeded in the opposite channel. The cells are grown to confluence and fluid flow is introduced into the BMEC compartment to create a blood-brain interface 146, 147. Usually, in these models, the pore diameter of PC membranes is 0.2 or 0.4 μm and the pore size of PDMS membranes ranges from 0.3 μm to 8 μm. Polyester (PE), polytetrafluoroethylene, and polyethylene terephthalate (PET) membranes have also been reported to support endothelial cells culture in microfluidic devices 74, 148, and these are optically transparent, making it easy to visualize cells by phase contrast microscopy. To assess the barrier function, Ag/AgCl or gold electrodes can be integrated into the chamber layer on opposite sides (blood and brain) of the porous membranes for real-time TEER measurements in the microfluidic system 74, 149. Wang *et al*. cocultured UM-RA BMEC-like cells with rat primary astrocytes in a BBB-on-chip model with integrated TEER electrodes. This system achieved TEER values above 2000 Ω·cm^2^ for up to 10 days 150. Vatine *et al*. created a BBB-on-chip with human UM-RA BMEC-like cells and neural progenitors. iPSC-derived neural progenitors supported the BBB maturation with TEER value over 1000 Ω·cm^2^ for 5 days. This system permits whole blood perfusion to the vascular lumen and protects neural cells from blood induced cytotoxicity. Additionally, patient-specific iPSC-derived BBB-on-chip models may predict the drug permeability for drug screenings 151. However, in these models, the cell-cell contact is restricted by the pore size and stiffness of membrane and rectangular structure of microchannels. Furthermore, due to the separated channel height, image acquisition in high resolution is challenging.

To mimic realistic vascular geometry, cylindrical tubular microvessels are another popular strategy to fabricate BBB models (Figure 2D). Microvascular tube structures can be constructed by inserting microneedles 152, glass rods 111, or nitinol wire 134, 153 into gel matrix prior to polymerization. After the gel has polymerized, the insert is removed and a cylindrical microchannel remains. Cells can be flowed into the microvessel and allowed to attach and form a monolayer around the sides. This microvessel platform enables controlled blood flow through the BMEC lumen encased by ECM in which pericytes and other neural cells reside to mimic the physiological microvascular structures. These microvessel models have been reported to exhibit robust physiological barrier functions. 150 μm-diameter microvessels were formed by encapsulating iPSC-derived BMEC-like cells in a rat tail type I collagen hydrogel. By crosslinking the matrix with genipin, the stiffness of gels was manipulated, ranging from 0.3-3.3 kPa. Matrix stiffness is well known to affect the adhesion and spreading of BMECs 134. Although the matrix stiffness did not significantly change the permeability of CHIR-RA BMEC-like cells in microvessels, the dilation response showed an increasing linear trend with increased transmural pressure and a dependence on matrix stiffness 153. In another study, E6 BMEC-like cells were assembled in a porcine gelatin crosslinked 3D channel and retained stable barrier function (measured by efflux transporter activity) for up to 3 weeks under different shear stresses 154. In these models, ECM gels can incorporate various cell types to form a biomimetic 3D BBB structure. Cell-cell interactions are more sufficient which facilitate the formation of basement membrane. The stiffness of the ECM can be controlled to mimic the health or disease condition since the stiffness of brain matrix changes in numerous neurological diseases 155-157. However, it is difficult to integrate the TEER measurements due to the cylindrical structure of these on-chip systems.

Building upon the above noted systems in order to mimic a more complex neurovascular unit, multiple parallel channels have been fabricated to assess the influence of blood vasculature with neural cells in 3D ECM gels (Figure 2E). In one system, ECM gels formed the channel by trapezoidal or phaseguide structures, which can guide the formation of the ECM gel and prevent gel flowing into the adjacent channels 158, 159. The flanked channel allowed the BMECs to form the blood vessel structure 159. Based on these designs, multiple BBB units were integrated into one microfluidic device to facilitate the high-throughput BBB assay. Xu *et al*. created a device that contained 16 independent functional BBB units connected by a microchannel network. Each BBB unit consisted of four BBB regions, each of which consisted of one vascular channel and one parallel channel for ECM collagen or astrocytes 160. Wevers *et al*. used OrganoPlates, a commercially available microfluidic BBB platform which harbor 40 three-lane or 96 two-lane chips in 384-well plate 161. These integrated devices make it possible to manipulate shear stress, cell types, nutrient delivery, and drugs to the vascular or brain compartments in a high-throughput manner.

To better recapitulate the physiological features of the BBB, self-organized microvascular networks were generated to mimic the natural processes of angiogenesis 162(Figure 2F). Endothelial cells sprout from preexisting vascular channels and self-assemble into tubular structures in adjacent ECM gels 163, 164. The resulting microvessels form intact and perfusable capillaries and exhibit native physiological morphologies. This BBB model allows for high-resolution imaging of key events at the BBB, such as cancer cell extravasation 165. Incorporation of pericytes and astrocytes assists the ECs in forming smaller (diameters ranging from 10 to 50 μm) and more branched vascular networks with decreased permeability values and upregulated BBB transporters 166. One limitation of this model is the difficulty to integrate the TEER measurement; however, we reiterate that TEER is likely not the best method for assessing and comparing BBB models. An additional limitation is that the fibrinogen hydrogel commonly used in this self-assembly model does not fully recapitulate the brain ECM. Meanwhile, the ability to continuously perfuse fluid through these model microvascular networks is an attractive feature and should be incorporated in more future studies 167.

#### 4.4.2 Shear stress in microfluidic BBB models

Shear stress is generated by blood flow and acts tangentially on the endothelial surface of blood vessels. Shear stress is a key mechanical cue that is critical in maintaining a stable BBB phenotype. Shear stress not only alters cellular morphology and differentiation of BMECs but can also trigger biochemical and biological events 169. In the BBB, BMECs regulate the transport of solutes and water between blood and brain tissues by sensing the shear stress. The physiological shear stresses range from 1-4 dyn/cm^2^ in venous systems to 10-20 dyn/cm^2^ in capillaries 168. Physiological shear stress applications result in an elongated spindle-like morphology and alignment of peripheral endothelial cells in the direction of flow. In contrast to peripheral endothelial cells, primary BMECs resist elongation and alignment in response to shear stress and maintain their cobblestone-like morphology 111, 170. Consistent with primary BMECs, iPSC-derived BMEC-like cells do not elongate and align upon exposure to shear stress 168. Meanwhile, shear stress decreased the proliferation, apoptosis, and cell displacement of iPSC-derived BMEC-like cells but did not affect the expression of key BBB markers in a microfluidic model 168. Shear stress enhances the integrity and stabilizes the barrier function of iPSC-derived BMEC-like cells. Several BBB models demonstrated that shear stress increased the TEER value 150, 151. A recent microfluidic system achieved physiological relevant TEER values by coculturing UM-RA BMEC-like cells with iPSC-derived neural progenitors 151. In a perfused hydrogel model, E6 BMEC-like cells were 10-100 times less permeable than HUVECs and primary BMECs. E6 BMEC-like cells exposed to shear stress (1 and 3.2 dyn/cm^2^) for 14 days that went through angiogenic sprouting and reduction of passive barrier function displayed a measured permeability value much lower than E6 BMEC-like cells cultured in static conditions 154. Physiologic shear stress protects the BMECs from inflammatory cytokines, while abnormal flow patterns impair barrier function of BMECs. Meanwhile, loss of flow induced TNF-α release, which decreased the expression of occludin, claudin-5, and VE-cadherin in BMECs and increased BBB permeability 171, 172. However, high shear stress (40 dyn/cm^2^) or pulsatility also decreased the expression of tight junction markers 173. Thus, maintaining the shear forces at physiologically relevant conditions is very important to stabilize BBB function.

## 5. BBB disease modeling

BBB dysfunction has been observed as a feature of various neurological diseases, including PD, AD, Huntington's disease (HD), and amyotrophic lateral sclerosis (ALS) 174. hiPSCs could be harnessed as powerful tools to recreate functional NVUs which provide a promising route to reconstruct functional BBBs *in vitro*. Beyond mimicking BBB physiological function, isogenic and patient-customized models have great promise to replicate complex disease processes such as progression of neurological diseases, brain metastases, and CNS infections. These applications also lend themselves useful for drug screening for novel treatments of these brain diseases. A cerebral ischemia model was created with iPSC-derived BMEC-like cells by inducing an oxygen-glucose deprivation (OGD) condition. TNFα was found to prevent the restoration of barrier integrity in OGD induced ischemia 80. E6 BMEC-like cells from patients with genetic neurological diseases show compromised barrier functions; for example, they carry mutations in PARK2, a PD early onset gene, which leads to loss of Pgp function in an apical-basolateral transport assay 70. Familial AD mutations (presenilin1 and presenilin2) display impaired barrier properties and glucose metabolism which are associated with the β-amyloid (Aβ) deposition in AD iPSC-derived BMEC-like cells 175, 176. Also, apolipoprotein (APOE4) is the strongest risk factor for sporadic AD. APOE4/4 iPSC-derived CHIR-RA BMEC-like cells, pericytes, and astrocytes self-assembled in Matrigel, forming capillary-like structures. APOE4 BBB models show AD vascular pathology upon increased Aβ accumulation, which is attributed to the dysregulation of nuclear factor of activated T cells-calcineurin signaling in APOE4/4 iPSC-derived pericytes 177. Additionally, UM-RA BMEC-like cells of HD patients manifest cell-autonomous deficits including impaired MDR1 function, transcytosis, and altered gene networks of barrier and angiogenesis 81. The expression of junction protein (Claudin-5) and TEER values were also significantly decreased in BMEC-like cells derived from iPSCs of HD patients 71. The monocarboxylate transporter 8 (MCT8)-deficient UM-RA BMEC-like cells derived from iPSCs of psychomotor retardation patients exhibited no significant differences in TEER and fluorescein permeability but showed reduced triiodothyronine (T3) permeability. The restoration of T3 transport can be explored as a potential to screen for drugs to treat MCT8-deficient patients 178.

Microfluidic BBB-on-chips permit the recreation of multicellular BBB architectures by incorporating multiple iPSC-derived cells. Personalized BBB-on-chips incorporating iPSC-derived UM-RA BMEC-like cells, astrocytes, and neurons exhibited physiological relevance with low paracellular permeability, high TEER value, response to inflammatory cues (IL-1β, IL-8, TNF-α), active transferrin RMT, and efflux transport. BBB chips from MCT8 HD and psychomotor retardation patients treated with whole human blood perfusion mimic multiple disease features and can be used to predict CNS drug penetrability 151. Meanwhile, a recent study showed that a BBB-on-chip allowed for more rapid evaluation of nanoparticle permeability, which could potentially predict nanoparticle transport and contribute to screening of nanotherapeutics 179. In addition, CRISPR/Cas9-mediated genome editing in patient-derived iPSCs allows for precise therapies targeted to inherited neurological diseases. Correction of mutants in HD and MCT-8-deficient iPSCs restores the barrier function in BBB models 153. In brain metastasis, malignant tumor cells have the ability to transmigrate through the BMECs of brain capillaries to enter into brain. Lung cancer, breast cancer, and malignant melanoma contribute to the majority of brain metastases 180. A high-throughput BBB-on-chip reproduced the process of tumor cell extravasation across the BBB by perfusing various cancer cell types through the vascular compartment 160. Lastly, to date, the exact mechanism of how the pathogens such as viruses and bacteria cross the BBB and enter the CNS is still largely unknown. Kim *et al*. showed that group B *Streptococcus* (GBS) invaded UM BMEC-like cells and activated cells to upregulate the proinflammatory chemokines and cytokines which contribute to the disruption of the tight junction components 82. Thus, iPSC-derived BBB models could uncover the infection process of pathogens within CNS.

## 6. Challenges and perspective

Thanks to the advances in human PSC-based technologies, a series of brain cell types derived from PSCs have been able to pave the way for superior BBB modeling. Significant efforts have been made to reconstruct BBB structure in 2D or 3D models which have incorporated the typical BBB features and have been utilized for various applications such as disease modeling, drug screening, and personalized medicine. Microfluidic BBB-on-chip models show huge potential to recapitulate more complex structure and function of *in vivo* BBB than conventional 2D BBB models which provide a more promising tool to facilitate mechanobiological study and drug discovery. Moreover, BBB models established from healthy or diseased donors could lead to more effective personalized therapies or novel drugs. Although PSC-derived BBB models have developed rapidly, their clinical applications are still at an early stage.

The ideal BBB model should reproduce the sophisticated brain structures and function which involve multiple determining factors such as cell invasion and migration, cell-cell interaction, controllable fluidic flow, and biomimetic microenvironment. The major challenges of BBB modeling are gaps between the *in vitro* model and *in vivo* capillary structure. Human brain perforating capillaries (the most abundant type) can be as small as 5-8 μm in diameter, and the inter-capillary distance is around 40-60 μm 21, 181. Most current approaches for modeling the BBB in microfluidic devices involve creating microchannels around 75-100 μm, which is closer to the size of arterioles or post-capillary venules 1. Self-assembled microvessels can achieve smaller capillaries with diameters of around 25-30 μm 166.

The BBB traits are generated by a dynamic interplay with multiple cell types including BMECs, pericytes, astrocytes and other neural cells. Primary BMECs lose their superior barrier properties when cultured *in vitro*. The current hPSC-derived BBB models are dependent upon the heterogeneous incorporation of hPSC-derived BMECs, pericytes and astrocytes. hPSC-derived BMEC-like cells recapitulate many functional and molecular features of *in vivo* BMECs and significantly improve our understanding of BBB development and functions 66. However, restricted by the differentiation methods, many models lack some phenotypic and functional aspects of *in vivo* BMECs, such as the key adhesion molecules involved in immune cell migration, some transporter activity, and responses to inflammatory stimuli 9, 74. Transcriptomic analysis highlights that epithelial-like genes are expressed in the hPSC-derived BMEC-like cells. It has been suggested that the most current protocols for BMECs differentiation produce a more homogenous epithelial cell population instead of endothelial cells 76, 79. Although transcriptomic profiles may not fully match the proteome expressed in BMECs, these results raise the questions of whether the epi-BMEC-like cells are suitable for use as *in vitro* BBB models and if these models are physiologically relevant and predictive of the *in vivo* situation. Meanwhile, many studies support that the hPSC-derived BMECs possess multiple of the requisite BBB markers and phenotypes, such as strong barrier properties, and a new model even captures relevant immune phenotypes 66. Continued efforts are required to develop homogenous BMECs for stable and reliable BBB models.

Although hPSC-derived BMECs, pericytes and astrocytes are usually included in BBB models *in vitro*, cells derived from different protocols are not well evaluated in the BBB models. How cell origins, such as hPSC mesoderm- or neuroectoderm-derived BMECs and pericytes affect the function of BBB models is still under-researched. Beyond assembling BMECs, pericytes, and astrocytes together, a combination of other NVU cell types, such as neural cells, may contribute to more comprehensive microenvironments and functionality of BBB models. Generally, neurological diseases exhibit brain vascular pathologies associated with changes in different brain regions, which may lead to different neuro-pathologies 40. The current iPSC-derived BBB models are simple assemblies of vascular associated cells and neuronal cells in different channels or gels without forming sophisticated 3D structures specific to particular brain regions. Region-specific BBB-associated pericytes, astrocytes, and neuronal cells have not been fully identified. Even in the current simplified BBB models, the direct and/or paracrine interaction of different cell types is still under-researched. It is important to note that hPSC-derived microglia or oligodendrocytes are missing in most of the current BBB model constructions which may have further effects on BBB function. The microfluidic BBB vasculature combined with cerebral organoids in a 3D manner may give more insights into these questions 182. In addition, age-related shifts in BBB dysfunction allow neurotoxic proteins to enter the aged parenchyma, which can trigger neuroinflammation and provoke neurodegenerative disease. Using iPSC-derived cells to recapitulate the BBB in an aging brain is still proving to be a challenge. Furthermore, recent work has identified that sex of cells is an important biological parameter that impacts neurodegenerative disease and BBB integrity, though most studies fail to address these discrepancies 183. Hence, comprehensive studies are still necessary to develop *in vitro* models that fully reflect the physiological functions of the BBB, such as the exchange of molecules, cell trafficking, and immune responses in different scenarios (age, sex, or disease-related conditions).

The mechanical cues from underlying matrix have been documented to influence the BBB properties^184^. A range of materials (collagen I, collagen IV, fibronectin, laminin, and agrin, etc.) have been explored with the aim of reproducing physical cues for the BBB *in vitro*. However, incorporation of multiple characteristics (cell-cell interaction, cell-matrix interaction, physiological shear stress, etc.) in 3D BBB models remains challenging. The materials with lower Young's Moduli, high pore densities, and the capability of recapitulating sophisticated 3D structure and interactions of the BBB are highly desired 185. In addition, long-term maintenance of BBB properties under dynamic fluid flow is still a challenge. Flow-mediated shear stress can promote the barrier properties of BBB models. The long-term culture of BBB models will be necessary for the study of chronic and age-associated diseases such as multiple sclerosis, PD or AD. So far, the longest successful culture of a microfluidic PSC-derived BBB model is around 3 weeks 154. The barrier function decreased over time which was also associated with different shear stresses applied to the flow system. Moreover, in most cases, the fluidic flow is perfused by culture medium, whereas whole blood perfusion could better mimic the *in vivo* physiological scenario. The optimized cultured condition is crucial to maintain a long-term culture. How to stabilize the barrier properties under a wide range of physiological blood flows still needs to be determined in the future studies. Furthermore, to meet the needs of the pharmaceutical industry, *in vitro* BBB systems should be stable, fully scalable, high throughput, and customizable with high predictability and reproducibility. These will require the incorporation of a number of automations such as real-time monitoring and control of physiological parameters, medium sampling, and data analysis, which will greatly minimize variability and facilitate operator efforts.

Despite significant advances in generating various hPSC-derived NVU cells and engineering novel BBB models, it is still challenging to incorporate all the relevant factors such as different NVU cells, ECM, and mechanical cues in one BBB model. The complexities and the limitation of different BBB models need to consider for the goals of the experiments. One needs to keep in mind that the reduced complexities of the BBB modeling would not fully recapitulate the function of BBB and compromise the outcomes. Therefore, the field should be continuing to work towards generating the full experimental insights that could be used to identify, in a systematic way, what *in vitro* model characteristics are sufficient to generate informative and relevant data.

## Figures and Tables

**Table 1:** Representative studies for hPSC-derived BMECs

Cell lines	Initial stage	BMEC specification	Markers	Functional assessment	Reference
IMR90-4, DF6-9-9T, DF19-9-11T, H9	Neural and endothelial co-differentiation	UM, hESFM, bFGF, PDS	PECAM-1, Claudin-5, GLUT-1, Pgp, VE-cadherin	TEER: ~850 Ω_cm^2^ LDL uptake Permeability: radiolabeled small molecules Efflux transporter activity: Pgp, MRP, BCRP Tube forming Co-culture with rat astrocytes	67
IMR90-4, DF19-9-11T, H9	Neural and endothelial co-differentiation	UM, hESFM, bFGF, PDS, RA	GLUT-1, Claudin-5, Occludin, PECAM-1, VE-cadherin	TEER: ~5000 Ω·cm^2^ Permeability: radiolabeled sucrose Efflux transporter activity: Pgp, MRP, BCRP Tight junction quantification: Occludin, Claudin-5 Co-culture with human primary pericytes, neural progenitor cells, and foreskin fibroblasts	68
IMR90-4	Neural and endothelial co-differentiation	UM, hESFM, bFGF, PDS, RA	PECAM-1	OGD induced *in vitro* model of cerebral ischemia TEER Permeability Cytokine treatment	80
CS83iCTR33n1, CS14iCTR28n6, CS21iHD60n8, CS04iHD66n4, CS81iHD71n3, CS09iHD109n1	Neural and endothelial co-differentiation	UM, hESFM, bFGF, PDS, RA	PECAM-1, GLUT-1, Claudin-5, Occludin, ZO-1	TEER: decreased in HD BMEC-like cells Efflux transporter activity: Pgp, ABCB1 Transcytosis: albumin Wound healing assay	81
DF19-9-11	Neural and endothelial co-differentiation	UM, hESFM, bFGF, PDS, RA	Claudin-5, Occludin, ZO-1	GBS infection assay TEER	82
IMR90-4, DF19-9-11T, H9	Primitive streak-like stage	CHIR99021, bFGF, RA, B27, hESFM,	PECAM-1, ZO-1, VE-cadherin, GLUT-1, Claudin-5, Occludin, BCRP, MRP, Pgp, vWF	TEER: above 3000 Ω·cm^2^ LPL uptake Efflux transporter activity: Pgp, MRP, BCRP Coculture with primary pericytes, hPSC-derived neurons and astrocytes.	73
IMR90-4, CD12, SM14, CC3	Neuroectoderm	E6, hESFM, bFGF, PDS, RA	GLUT-1, Claudin-5, Occludin, PECAM-1, VE-cadherin	TEER: above 2500 Ω·cm^2^ Permeability: sodium fluorescein Efflux transporter activity: Pgp, MRP Co-culture with human primary pericytes and iPSC-derived astrocytes	70
CC3, CD10, HD70-2, and TSP8-15	Neuroectoderm	E6, hESFM, bFGF, RA, PDS, B27, ITS	PECAM-1, Claudin-5, GLUT-1, VE-cadherin, Occludin	TEER: ~3000 Ω·cm^2^ Permeability: sodium fluorescein Efflux transporter activity: Pgp, MRP Tight junction quantification: Occludin, Claudin-5 Co-culture with human iPSC-derived astrocytes	71
IMR90-4	Neural and endothelial co-differentiation	Hypoxia: UM, hESFM, bFGF, PDS, RA	ZO-1, Claudin-5, PECAM-1, GLUT-1, Pgp	TEER: 25000 Ω Efflux transporter activity: Pgp, MRP1, MRP4, BCRP Permeability: 3, 10 kDa dextran	74
IMR90-4, H1, H6	Neural and endothelial co-differentiation	UM, hESFM, bFGF, PDS, RA, transduction of *FL1*, *ERG*, and *ETV2* lentiviral vectors at D6	ZO-1, Occludin, PECAM1, CDH5, EPCAM-1	TEER: ~200 Ω·cm^2^ Permeability: 70 kDa dextran Formation of a capillary network Responses to TNFα	76
CC3, CD10, CDH5-2A-eGFP	Neuroectoderm	E6, hESFM, DMEM/F12, neurobasal, bFGF, RA, B27	GLUT-1, Claudin-5, Occludin, VE-cadherin	TEER Permeability: sodium fluorescein Efflux transporter activity: Pgp, MRP	72
IMR90-4, iPSC donor 1, 2, and 3.	CD34^+^CD31^+^ EC progenitor cells	LaSR, hESFM, B27, bFGF, RA	Claudin-5, Occludin, VE-cadherin, PECAM-1, ZO-1	TEER Permeability: sodium fluorescein Pro-inflammatory cytokine simulation Co-culture with iPSC-derived smooth muscle-like cells, human astrocytes, hiPSC-derived astrocytes, bovine pericytes, and human brain pericytes. Tube forming in mouse	9

**Table 2:** Representative studies for hPSC-derived pericytes

Cell lines	Initial stage	Pericytes specification	Markers	Functional assessment	Reference
H9.2, I6, C3, KTR13	Spontaneously differentiation EBs	EBM-2, EC M-19, FBS	CD105, CD90, CD73, CD31, CD146, NG2, and PDGFR positive	Pericytes and EC assembly Hind limb ischemia model	90
H9, H13, clone 26 hCBiPS aMHC^neo^PGK^hygro+^, pCAGGS2, hiPSC-MR31, hESC-H9, hiPSC-BC1	Early vascular cells 12d Pericyte inducing condition 6d	EVC: CIV serum VEGF, SB431542 in EC media Pericytes Serum aMEM	CD73, NG2, PDGFRβ, CD44 positive, VE-cadherin and CD31 negative	Self-organized vascular networks in collagen and HA hydrogels *In vivo* integration	94, 95
HESC-NL4, Fib-iPSC BOEC-iPSC, NL-HES4, HES3 (NKX2-5eGFP/w)	CD31- fraction	Activin A, BMP4, CHIR99021, VEGF, SB43152 DMEM, FBS, TGFbeta3, PDGF-BB	PDGFRβ, CD146, NG2, CD73, CD44, CD105	Vascular Plexus Zebrafish xenografts	91 92
ESI-017	EB	BMP4, FGF2, Activin-A, VEGFA, SB431542	CD146, CD73, and CD105 positive; CD31, CD34, and CD133 negative	Tube formation assay	99
H1, H9-EGFP, IISH2i-BM9	Mesenchymal progenitors Immature pericytes	FGF2, PDGF-BB, SB431542, VEGF, EGF	Capillary Phenotype: NG2^+^ α-SMA^low/**-**^Desmin^low/**-**^Calponin^ low/**-**^ MYH11**^-^** Arteriolar phenotype: NG2^high^ α-SMA^+^ Desmin^+^ Calponin^low/**-**^ MYH11^-^	Vasculature formation *in vitro* and *in vivo*	96
BC1, C12-RFP	Mesoderm, early vascular, pericyte maturation	sB431542, VEGF, pericytes medium	PDGFRβ, NG2, CD31, Calponin	Transwell: coculture pericytes with hPSC-derived BMEC-like cells	100
AD5, AD6, AD13, AD14, AD20, AD22, AD29, H9 (WA09) and H1 (WA01)	Mesoderm Neural crest	Mesoderm: MIM, DKK1, pericytes medium Neural crest: B27, CHIR99021, pericytes medium	PDGFRβ, NG2, CD13, and CD146 positive	Transwell: coculture pericytes with hPSC-derived BMEC-like cells	97
H9, IMR90C4, CS03n2	Neural crest	E6, CHIR99021, SB431542, bFGF, dorsomorphin, FBS	PDGFRβ, NG2	Self-assembling with endothelial cells Transwell: coculture pericytes with iPSC-derived BMEC-like cells	98
H1, H9, DF19-11, 005B23.1, CD3-3, PMBC-3-1, WTC11, WT83, Q83X, M2	Mesoderm CD34^-^ fraction	E8BAC medium: E8, BMP4, Activin-A, and CHIR99021 E7BVi: E8 minus TGFβ1, BMP4, VEGFA, and SB431542	CD34, CD31 negative, PDGFRβ, α-SMA, SM22 positive	Angiogenesis assay	93

**Table 3:** Representative studies for hPSC-derived astrocytes

Cell lines	Initial stage	Astrocyte specification	Markers	Functional assessment	References
H9, H7, IMR90-4	Neuroepithelia	RA, FGF8, SHH, CNTF, LIF, FBS	S100β, GFAP	glutamate uptake synaptogenesis Calcium wave Astrocyte-neuronal co-culture Animal transplantation	103
H9, HUES9	bFGF, EGF	B27, BMP4, LIF	S100β, GFAP, EAAT1, aquaporin	Oxidative neuronal injury	104
WA-09, WA-01, IMR90- 4, iPS-Foreskin-1	N2, bFGF, FGF, EGF, CNTF,	N2, CNTF	S100β, GFAP	Migratory capacity Tropism for hHGG	105
H9, H14, BC1	B27, bFGF, CNTF, BMP	CNTF, BMP, FBS	GFAP, TUJ1	Integrate *in vivo*	106
N116213, N117322, 409B2, APP1E111, APP1E211, APP1E311, APP2E22, APP2E26, AD3E211, AD8K213	Cortical neuron: N2, SB431542, dorsomorphin, B27, BDNF, GDNF, NT-3	Repeat passage to a non-coated polystyrene dish	GFAP	Accumulation of Ab oligomers	107
4.2 line, GM003814 unaffected 21.8 line, GM002183; SMA 3.6 line, GM003813; SMA 7.12 line, GM09677	bFGF, EGF,	B27, CNTF	GFAP	Disease phenotypes of SMA	108
M337V-1, M337V-2, CTRL-1 iPSC line, CTRL-2 iPSC line	LIF, EGF, bFGF,	B27, CNTF	S100β, GFAP	Astrocyte-neuronal co-culture Disease phenotypes of ALS	94
H9, SeV-derived iPSC line and modRNA-derived iPSC line	N2, bFGF, EGF, FGF, CNTF, Noggin, SB431542	CNTF	A2B5, GFAP	*In vitro* migratory capacity *In vivo* transplantation	109
HC1, HC2, HC3, MS1, MS2, MS3, MS4,	Noggin, bFGF, SB431542	bFGF, EGF, LIF, CNTF	S100β, GFAP, GLAST	Disease phenotypes of multiple sclerosis	110
WA09, H9, RRID:CVCL_9973,GM1-4, RRID:CVCL_7290	N2, B27, SB431542, DMH1	SHH, CHIR99021, CNTF, cyclopamine, prumorphamine, RA, bFGF, BMP4.	Regional markers: S100β, SOX9, GFAP, HEPACAM.	Basic membrane properties Co-culture with neurons and endothelial cells	102

**Table 4:** Representative studies for hPSC-derived microfluidic BBB-on-chip

Cell lines	Seed cells	Fluidic channel	Shear stress	Matrix	BBB markers	Function	Time of observation	Reference
BC1	UM BMEC-like cells	four rectangular channels with different heights	4 and 12 dyne/cm^2^	Collagen IV, Fibronectin	Occludin Claudin-5 F-actin ZO-1	Cell morphology, proliferation, apoptosis, protein gene expression under shear stress	40 h	168
IMR-90-4	UM-RA BMEC-like cells, Rat primary astrocytes	Neuronal Chamber: 6.5 mm diameter Microchannels: 300 μm width×160 μm height PC membrane: 0.4 μm diameter pores TEER electrode	0.023-1.8 dyne/cm^2^	Collagen IV, Fibronectin	Claudin-5, ZO-1	TEER: 2000-4000 Ω·cm^2^ Permeability: 4, 20, 70 kDa Dextran, caffeine, cimetidine, doxorubicin	10 days	150
BC1	UM-RA BMEC-like cells	Diameter: 150 μm	0.1, 1 dyne/cm^2^	Collagen I Laminin/entactin, Genipin, Collagen IV, Fibronectin	ZO-1, Claudin-5	TEER: transwell Permeability: 70 kDa Dextran	3 days	134
CS0617iCTR, CS0172 iCTR, CS0188 iCTR, CS81iHD, CS03iCTR, CS03iCTR^mut^, CS01iMCT8, CS01iMCT8^Cor^	UM-RA BMEC-like cells, primary pericytes and astrocytes	Brain channel: 1×1 mm Blood channel: 1×0.2 mm PDMS membrane: 7 μm diameter pores	0.01, 0.5, 2.4,5 dyne/cm^2^	Collagen IV, Fibronectin	Occludin Claudin-5 ZO-1, PECAM-1, GLUT-1	TEER Permeability: 3, 4, 20, 70 kDa Dextran T3, IgG, Albumin, Transferrin Efflux transporter activity: Pgp Viability: LDH	10 days	151
IMR90-4	Hypoxia induced UM-RA BMEC-like cells, human primary pericytes, and astrocytes	Brain channel: 2cm long×1mm wide×1mm high Blood channel: 2cm long×1mm wide 0.2 mm high PET membranes Pore size: 0.4 μm	6 dyne/cm^2^	Collagen IV, Fibronectin	ZO-1, Claudin-5, PECAM-1, GLUT--1, Pgp	TEER: 25000 Ω Efflux transporter activity: Pgp, MRP1, MRP4, BCRP Permeability: 3, 10 kDa dextran	2 weeks	74
BC1-GFP, C12-RFP	CHIR-RA BMEC-like cells	Diameter: 150 μm	1 dyne/cm^2^	Collagen I, Genipin, Matrigel	ZO-1	Permeability: Lucifer yellow, 10 kDa dextran	2 days	153
IMR90-4, CC3	E6 BMEC-like cells, HUVEC, μVas	Diameter: 800 μm	0.3, 1, 3 dyne/cm^2^	Gelatin	Occludin Claudin-5 VE-cadherin F-actin	Permeability: 3 kDa Dextran, Albumin Efflux transporter activity: Pgp, MRP	21 days	154
	iPSC-ECs, human primary pericytes, and astrocytes	Self-organized vessels Diameter: 10-200 μm	Not identified	Fibrin gel	ZO-1, Occludin Claudin-5	Permeability: 10 kDa and 40 kDa dextran	7 days	166

## Abbreviations

ABC: ATP-binding cassette
AD: Alzheimer's disease
ALS: amyotrophic lateral sclerosis
ANG: angiopep-2
BBB: blood-brain barrier
BCRP: breast cancer resistance protein
BDNF: brain-derived neurotrophic factor
bFGF: basic fibroblast growth factor 2
BMECs: brain microvascular endothelial cells
BMP: Bone morphogenetic protein
CNS: central neuron system
CDH5: cadherin 5
CNTF: ciliary neurotrophic factor
DMH1: dorsomorphin homologue 1
EB: embryo body
EBM-2: endothelial cell growth basal medium-2
ECs: endothelial cells
ECM: extracellular matrix
EGF: epidermal growth factor
EPCAM: epithelial cell adhesion molecule
ERG: E-twenty six-related gene
EGF: epidermal growth factor
ETV2: E-twenty six variant 2
FBS: fetal bovine serum
FL1: friend leukemia integration 1 transcription factor
GDNF: glial cell line-derived neurotrophic factor
GFAP: glial fibrillary acidic protein
GLAST: glutamate transporter
GBS: Streptococcus agalactiae
GLUT-1: Glucose transporter 1
HA: hyaluronic acid
HD: Huntington's disease
hESC: human embryonic stem cell
hESFM: human endothelial serum-free medium
hHGG: human high-grade gliomas
ICAMs: leukocyte adhesion molecules
iPSC: induced pluripotent stem cells
ITS: insulin, transferrin, and selenium
LDL: low-density lipoprotein
LIF: leukemia inhibitory factor
LPR1: low-density lipoprotein receptor-related protein
MIM: mesoderm induction medium
MRP: multidrug resistance associated protein
MSC: mesenchymal stem cells
NPCs: neural progenitor cells
NT-3: neurotrophin-3
OGD: oxygen-glucose deprivation
PC: polycarbonate
PET: polyethylene terephthalate
PSC: pluripotent stem cell
NVU: neurovascular unit
PDGF-BB: platelet-derived growth factor-BB
PDS: platelet-poor plasma-derived serum
PECAM-1 (CD31): platelet endothelial cell adhesion molecule-1
Pgp: P-glycoproteins
RA: retinoic acid
RMT: receptor mediated transcytosis
RMT: receptor mediated transcytosis
SHH: sonic hedgehog
SMA: spinal muscular atrophy
TfR: transferrin receptor
TGF: transforming growth factor
TNFα: tumor necrosis factor alpha
VEGF: vascular endothelial growth factor
ZO-1: zonula occulden-1
